# Immediate Changes in Physical Function, Psychological Health, and Health-Related Quality of Life Following a Five-Day Multicomponent Coastal Health Program in Postmenopausal Women: A Randomized Controlled Trial

**DOI:** 10.3390/jcm15155926

**Published:** 2026-07-29

**Authors:** Sung-Hyeon Kim, Jin-Hwa Jung, Ji-Eun Baek, Ho-Jin Shin, Hwi-Young Cho

**Affiliations:** 1 Department of Physical Therapy, Gachon University, Incheon 21936, Republic of Korea; gpgkorea30@gmail.com (S.-H.K.); baekjieun421@gmail.com (J.-E.B.); 2 Department of Occupational Therapy, Semyung University, Jecheon 27136, Republic of Korea; otsalt@semyung.ac.kr; 3 Wellness Center, Ansan University, Ansan 15328, Republic of Korea

**Keywords:** postmenopausal women, multicomponent intervention, coastal environment, nature-based intervention, physical function, health-related quality of life, perceived stress, depressive symptoms, mindfulness meditation, randomized controlled trial

## Abstract

**Background/Objectives**: After menopause, hormonal, age-related, and lifestyle factors may adversely affect physical functioning and psychological well-being. This study compared immediate changes in physical function, psychological health, and health-related quality of life following a five-day Combined Marine Treatment Program (CMTP) with those observed under a usual-routine control condition. **Methods**: A parallel-group randomized controlled trial was conducted with 62 postmenopausal women allocated equally to the CMTP and control groups. The CMTP included coastal walking, resistance exercises, mindfulness meditation, and visits to local natural attractions. Participants in the control group continued their customary daily activities. The co-primary outcomes were the Timed Up and Go test and the Short Form-36. Secondary outcomes were grip strength, pinch strength, static balance, the 10-item Perceived Stress Scale, and the Beck Depression Inventory-II. Mixed repeated-measures analysis of variance was used to test whether temporal changes differed between groups. Trial registration was completed through the Clinical Research Information Service of the Republic of Korea (KCT0009741). **Results**: Changes over time differed significantly between groups for the Timed Up and Go test, grip strength, pinch strength, eyes-open sway area, eyes-closed sway area, eyes-closed sway velocity, and all self-reported outcomes. Post-intervention values differed significantly between groups for the Timed Up and Go test, pinch strength, both eyes-closed balance measures, and all self-reported outcomes. No adverse events were reported during the trial. **Conclusions**: Relative to the usual-routine condition, the five-day CMTP was associated with immediate changes in selected performance-based physical outcomes and broader changes across self-reported psychological and health-related quality-of-life outcomes. Brief multicomponent coastal health programs therefore warrant further study in postmenopausal women.

## 1. Introduction

Menopause represents a midlife physiological transition shaped by hormonal and age-related changes [1]. Because women may live for several decades after menopause, the resulting changes can have sustained implications for health and daily functioning. Postmenopausal women may experience reductions in skeletal muscle mass and hand strength, together with broader musculoskeletal and physical-performance limitations [2,3,4,5,6]. Women during and after the menopausal transition may also experience reduced quality of life and increased perceived stress, anxiety, and depressive symptoms [7,8,9,10,11,12,13]. Moreover, recent evidence indicates that depressive symptoms are associated with poorer mobility and walking performance in postmenopausal women [14], suggesting that physical and psychological health are interrelated rather than independent domains. Together, these concerns highlight the relevance of health strategies that address both physical function and psychological health.

Menopausal hormone therapy is the most effective option for vasomotor and genitourinary symptoms and may also reduce bone loss and fracture risk. Its use should nevertheless be individualized according to age, time since menopause, symptom profile, contraindications, formulation, dosage, administration route, and treatment duration [15]. Because hormone therapy is prescribed for specific clinical indications, complementary nonpharmacological strategies may also be relevant for supporting physical function, psychological health, and quality of life in postmenopausal women. Structured exercise, mind–body practices, and nature-based activities may be particularly relevant when multiple health domains are addressed within an integrated health-promotion program.

Among nonpharmacological approaches, exercise can address several functional concerns associated with menopause. Structured exercise has been shown to improve physical fitness and quality of life in postmenopausal women [16], while reviews of walking programs suggest potential benefits across physical and psychosocial health domains [17]. More recent evidence indicates that resistance, balance, and multicomponent exercise may improve balance-related outcomes in peri- and early postmenopausal women [18]. Mindfulness-based practices may complement these physical approaches by supporting attention regulation, body awareness, and stress management. A randomized trial reported an improvement in menopause-specific quality of life [19], and recent meta-analytic evidence suggests potential benefits for perceived stress, menopausal symptoms, and quality of life, although findings across psychological outcomes and intervention formats remain heterogeneous [20]. Together, exercise and mindfulness may provide complementary support for the physical and psychological health of postmenopausal women.

Natural environments may also provide a supportive context for exercise and mindfulness. Previous studies have examined nature exposure and forest-based programs in relation to health promotion and restorative responses [21,22]. More recent evidence suggests that outdoor blue-space exposure is positively associated with physical activity, perceived health, and mental well-being in older adults, although direct sensory exposure and causal evidence remain limited [23]. Randomized studies of nature walking and multicomponent nature-based programs further indicate that structured activity in natural settings may benefit affect and well-being [24,25]. Coastal activity programs have likewise shown potential psychological benefits; however, a trial comparing surf and hike therapy and a recent systematic review did not establish a consistent marine-specific advantage [26,27]. Coastal settings nevertheless offer diverse visual, auditory, and tactile experiences within which structured activities can be delivered, while sea–sand exercise has demonstrated feasibility in rehabilitation contexts [28]. Thus, coastal environments may provide a setting for integrated health programs combining physical activity, mindfulness, and structured exposure to nature.

However, randomized evidence remains limited regarding brief, supervised programs that integrate exercise, mindfulness, and nature-based activities in coastal settings and evaluate immediate changes in both physical function and psychological health among postmenopausal women [17,18,20,23,27]. Therefore, this study aimed to compare immediate pre–post changes in strength, balance, quality of life, perceived stress, and depressive symptom scores following a five-day Combined Marine Treatment Program (CMTP) with those observed under a usual-routine control condition.

## 2. Materials and Methods

### 2.1. Participants

This study recruited urban-dwelling postmenopausal women aged 40–69 years, with postmenopause defined as ≥12 months of amenorrhea. Participants were recruited via local newspaper advertisements and community center posters between March and June 2025, and the intervention was conducted from August to September 2025. The inclusion criteria [29] were as follows: (1) being recreationally inactive (<90 min/week of exercise), (2) not receiving hormone replacement therapy, and (3) being a non-smoker and not consuming more than two alcoholic drinks per day. The exclusion criteria [30] were as follows: (1) current use of antidepressants and (2) any unstable medical condition or physical incapacity that precluded participation in walking or resistance training. Before participation, each woman received information about the study purpose, procedures, anticipated benefits, and potential risks and subsequently provided written informed consent. Ethical approval was granted by the Institutional Review Board of Gachon University on 18 April 2024 (Approval No. 1044396-202312-HR-249-01). Trial details were entered into the Clinical Research Information Service (CRIS) in the Republic of Korea on 30 August 2024 (Registration No. KCT0009741), a registry participating in the World Health Organization International Clinical Trials Registry Platform.

The required sample was estimated a priori in G*Power (Version 3.1.9.4, Heinrich-Heine-University Düsseldorf, Germany) using the group × time interaction in a repeated-measures analysis of variance. Input parameters of f = 0.25, α = 0.05, 80% power, two groups, two measurements, a repeated-measures correlation of 0.50, and ε = 1.00 yielded a total requirement of 34 participants [31]. The minimum recruitment target was increased to 38 to accommodate 10% attrition, and 62 eligible and consenting women were ultimately randomized.

### 2.2. Study Design

**Figure 1:**
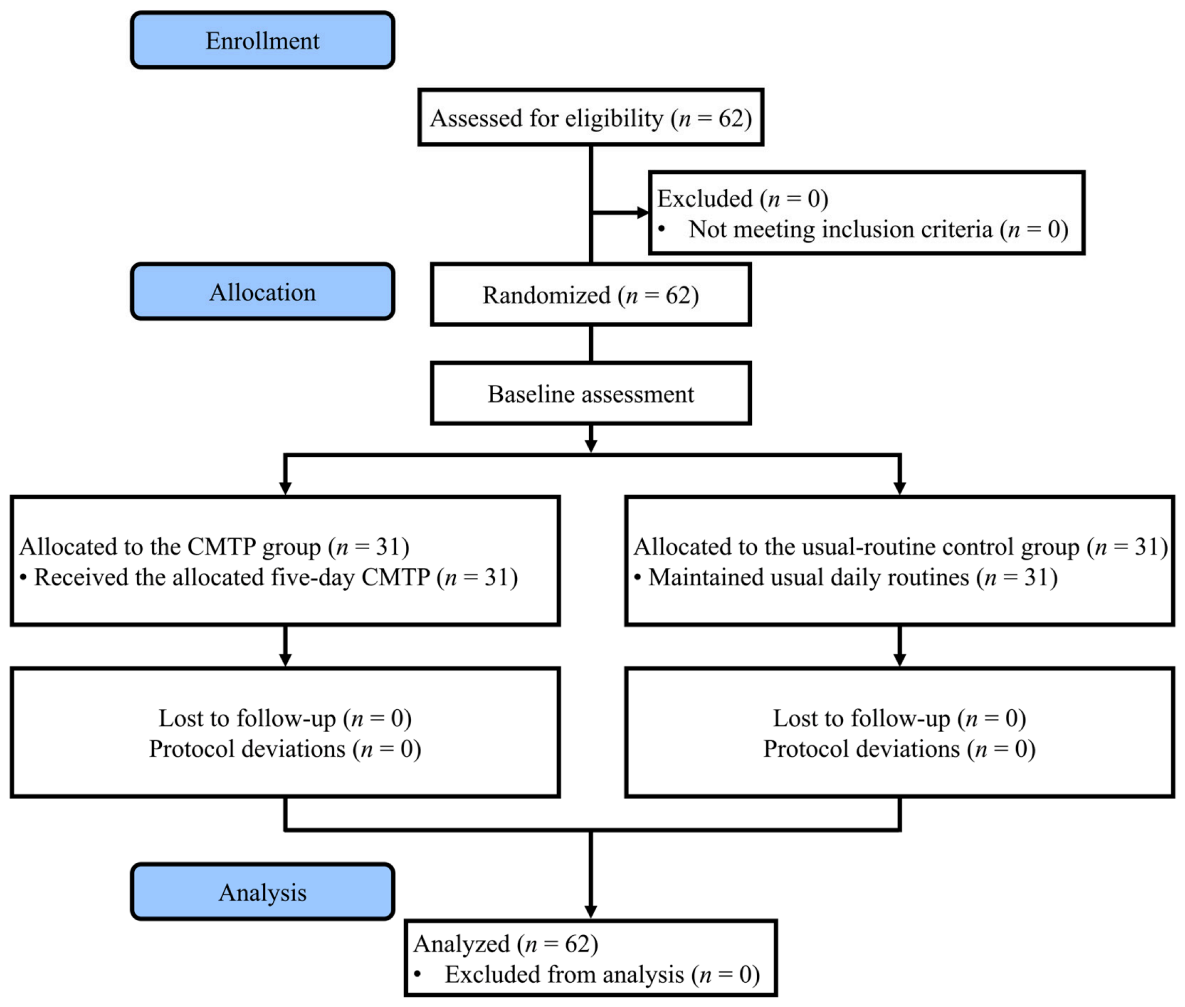
Flowchart of the study.

This study was a parallel-group randomized controlled trial (RCT) (Figure 1). Randomization was stratified by age, employment status, and education level. An independent statistician generated the computer-based allocation sequence and assigned participants to the Combined Marine Treatment Program (CMTP) group or the control group (CG) without involvement in intervention delivery or outcome assessment. The CMTP group began the program after the baseline assessment, whereas participants in the CG were informed that their health status during usual daily routines would be assessed before their scheduled participation in the CMTP. Because the groups followed different participation schedules, study procedures were conducted at separate locations and on different dates to minimize contact and information exchange between groups.

Participants assigned to the CMTP group completed the five-day supervised program, with pre-assessment conducted the day before the program and post-assessment conducted one day after completion of the final session. The CG maintained their usual daily routines during the same period, and both groups were assessed at the same time points. Licensed physical therapists with at least five years of clinical experience administered all questionnaires and assessments according to standardized protocols. The design and reporting followed the Consolidated Standards of Reporting Trials (CONSORT) 2025 guidelines for randomized controlled trials.

### 2.3. Study Site

The CMTP was conducted in Taean County, located on the Taean Peninsula along the western coast of South Chungcheong Province, Republic of Korea. Taean County is characterized by diverse coastal landscapes and sensory features, including beaches, coastal dunes, tidal flats, sea sounds, coastal winds, and beach formations, which formed the setting for the CMTP. Program activities were conducted in coastal areas and nearby natural attractions, including Chollipo Arboretum and the Sinduri Coastal Sand Dune, to provide structured exposure to coastal and other natural settings. Weather forecasts were reviewed in advance, and outdoor activities were scheduled during periods without anticipated weather-related safety concerns.

### 2.4. Combined Marine Treatment Program

The CMTP comprised three main components: physical activity, mindfulness meditation, and visits to local natural attractions (Table 1). Participant attendance and delivery of the scheduled program components were documented throughout the intervention period, together with any protocol deviations or adverse events. Physical activity sessions were supervised by a licensed physical therapist with a doctoral degree and more than 10 years of clinical experience, while visits to local natural attractions were guided by nationally certified nature guides.

#### 2.4.1. Physical Activity

Physical activity consisted of coastal walking and body recovery exercises. Participants completed 30 min of self-selected moderate-intensity coastal walking (approximately 3000 steps per session) at a target intensity of 50–60% of their age-predicted maximum heart rate. This walking activity was performed along the beach, providing exposure to the coastal environment [32]. Body recovery exercises comprised 40–50 min of stretching and resistance exercises designed to address flexibility, muscle strength, postural stability, and muscle relaxation [33,34,35]. Elastic-band resistance (TheraBand^®^; Performance Health, Downers Grove, IL, USA) was individualized according to each participant’s baseline strength and physical capacity. Bands with different resistance levels were available, and resistance was selected so that each participant could complete three sets of 10–15 repetitions with proper technique at a tolerable intensity. Because the intervention lasted five days, no predefined progression schedule was applied.

#### 2.4.2. Mindfulness Meditation

Mindfulness meditation was conducted in two formats: (1) coastal sensory meditation involving ocean views, sea breezes, and beach sand; (2) supine meditation intended to promote full-body relaxation [19,36]. An experienced instructor led each 30 min session, combining body scan meditation with controlled breathing to facilitate relaxation, attention regulation, and awareness of bodily sensations.

#### 2.4.3. Visits to Local Natural Attractions

Chollipo Arboretum provided exposure to ecological diversity and natural visual stimuli, whereas the Sinduri Coastal Sand Dune provided exposure to coastal topography and environmental features. Together, these visits constituted the nature-based component of the CMTP.

#### 2.4.4. Control Group Protocol

Participants in the control group (CG) continued their usual urban routines throughout the study period, without beginning structured exercise or additional meditation or visiting forest or coastal environments. Participants receiving ongoing medical treatment or medication were instructed to continue their usual regimens; any new treatment, medication change, or visit to a nature-based environment was recorded as a protocol deviation. Adherence to the control-group instructions was monitored using self-reported daily logs and regular telephone check-ins. After completing the post-assessment, participants in the CG were offered the same CMTP.

### 2.5. Outcome Measures

This study assessed physical function and psychological health using performance-based assessments and self-reported questionnaires. Physical function outcomes included the Timed Up and Go test (TUG), grip strength, pinch strength, and static balance. Self-reported psychological and health-related quality-of-life outcomes included the Short Form-36 Korean version (SF-36), the 10-item Perceived Stress Scale (PSS-10), and the Beck Depression Inventory-II (BDI-II). The prespecified co-primary outcome measures were the TUG test and the SF-36. The SF-36 was treated as a multidimensional primary measure, with its component summary and domain scores reported to characterize the response pattern rather than as separate primary endpoints. Secondary outcomes included grip strength, pinch strength, static balance, PSS-10, and BDI-II. All outcomes were assessed at baseline and immediately after the intervention period. Assessments were conducted at a designated research facility within the study site to ensure consistency in data collection.

#### 2.5.1. Dynamic Balance and Mobility

The TUG test, which has demonstrated an intraclass correlation coefficient (ICC) of ≥0.93 [37], was used to assess dynamic balance and mobility. From a chair with an approximately 46 cm seat height, participants stood at the verbal cue “go,” walked 3 m, turned, returned to the chair, and sat down. Completion time was measured from the cue until participants resumed the seated position, and the mean of three trials was analyzed.

#### 2.5.2. Grip and Pinch Strength

Grip and pinch strength were measured in kilograms using standardized protocols (grip strength ICC = 0.996–0.998; pinch strength ICC = 0.949–0.990) [38,39,40,41]. Grip strength was tested sitting with the hips and knees at approximately 90°, the shoulders adducted in neutral rotation, the elbows at 90°, and the forearms neutral. Participants applied maximal force while holding a Jamar hand dynamometer (Model 51731; Performance Health, Downers Grove, IL, USA) vertically. For pinch strength, maximal force between the thumb and index finger was recorded using a Jamar pinch gauge (Performance Health, Downers Grove, IL, USA). The highest of three trials was retained for each measure.

#### 2.5.3. Static Balance

Static balance was assessed using a force plate (AMTI Accusway system, Advanced Mechanical Technology, Inc., Watertown, MA, USA), which has demonstrated high test–retest reliability (ICC = 0.70–0.89) [42]. Force-plate signals were acquired at 200 Hz and processed with a fourth-order, 10 Hz low-pass Butterworth filter. During testing, participants crossed their arms over the chest and stood with a 30° foot angle and 9 cm between the heels while viewing an eye-level cross 1.5 m ahead. Three 30 s trials were completed under both visual conditions, separated by 60 s of rest, and the central 10 s segment of each trial was analyzed.

#### 2.5.4. Short Form-36 Korean Version

The SF-36 was used to assess health-related quality of life and daily functioning. It has demonstrated high reliability (Cronbach’s α = 0.93–0.94) [43,44]. The physical component was assessed using four subscales: Physical Functioning (PF), Role Functioning Physical (RP), Bodily Pain (BP), and General Health (GH). The mental component was assessed using four subscales: Vitality (VT), Social Functioning (SF), Role Functioning Emotional (RE), and Mental Health (MH). Scores range from 0 to 100, with higher scores indicating better health status.

#### 2.5.5. Perceived Stress Scale

Perceived stress was evaluated with the PSS-10, which examines how frequently situations are experienced as unpredictable, uncontrollable, or overwhelming. Its 10 items are scored from 0 (never) to 4 (very often), yielding a total of 0–40, with higher values reflecting greater perceived stress. The Korean version has demonstrated acceptable internal consistency (Cronbach’s α = 0.75) [45].

#### 2.5.6. Beck Depression Inventory-II

Depressive symptom severity was evaluated using the 21-item BDI-II. Item scores range from 0 to 3 and are summed to produce a total of 0–63, with higher totals representing more severe symptoms. The Korean version has demonstrated excellent internal consistency in Korean adults (Cronbach’s α = 0.946) [46].

### 2.6. Statistical Analysis

Data analyses were performed using Statistical Package for the Social Sciences (SPSS) software version 25.0 (IBM Corp., Armonk, NY, USA). Continuous variables were presented as means and standard deviations, and categorical variables as frequencies. No outcome measures were added or removed after the trial commenced. Data normality was evaluated with the Shapiro–Wilk test. Baseline group comparisons used independent-sample *t*-tests for continuous variables and chi-square tests for categorical variables. Intervention effects were examined using a mixed repeated-measures analysis of variance, with group (CMTP vs. CG) as the between-subject factor and time (pre- vs. post-intervention) as the within-subject factor. Post hoc pairwise comparisons of estimated marginal means were conducted for the within-group pre–post contrasts and the post-intervention between-group contrast, with Bonferroni adjustment applied within each simple-effect comparison family. Analyses of the secondary outcomes and individual SF-36 component and domain scores were exploratory. Interaction effect sizes were expressed as partial eta squared (η^2^p). All randomized participants were included in the final analysis, and no missing outcome data were observed. Statistical significance was set at *p* < 0.05.

## 3. Results

### 3.1. General Characteristics of Participants

All 62 randomized participants completed their assigned study procedures and were included in the final analysis. Attendance and program delivery were complete in the CMTP group, with no weather-related changes or cancelations. Daily logs and telephone check-ins identified no deviations from the usual-routine instructions in the control group. Neither group experienced adverse events or unintended effects. Participant characteristics are presented in Table 2.

### 3.2. Changes in Physical Function Outcomes After the Program

Table 3 summarizes changes in physical function outcomes, including the TUG test, grip strength, pinch strength, and static balance. Significant group × time interactions were observed for the TUG test, grip strength, pinch strength, SAEO, SAEC, and SVEC. Within the CMTP group, TUG completion time and all static balance measures decreased, whereas grip and pinch strength increased from baseline to post-intervention (all adjusted *p* < 0.05). At the post-intervention assessment, between-group differences were significant for the TUG test, pinch strength, SAEC, and SVEC (all adjusted *p* < 0.05). Within the CG, SVEC increased significantly, whereas the remaining physical function outcomes showed no significant pre–post changes.

### 3.3. Changes in Psychological Health and Quality-of-Life Outcomes After the Program

Table 4 summarizes changes in the SF-36, PSS-10, and BDI-II scores. Significant group × time interactions were observed for all SF-36 summary scores and subscales, PSS-10, and BDI-II (all *p* < 0.001). Within the CMTP group, all SF-36 summary and subscale scores increased, whereas PSS-10 and BDI-II scores decreased from baseline to post-intervention (all adjusted *p* < 0.001). At the post-intervention assessment, all self-reported outcomes differed significantly between groups (all adjusted *p* < 0.05), whereas no significant pre–post changes were observed within the CG.

## 4. Discussion

Compared with the usual-routine control condition, the five-day supervised CMTP was associated with immediate changes in the prespecified primary outcome measures of functional mobility and multidimensional health-related quality of life. Exploratory analyses of the secondary outcomes and individual SF-36 component and domain scores further indicated selective changes in performance-based physical outcomes and a broader pattern across self-reported psychological and health-related quality-of-life domains. Together, these findings suggest an immediate multidomain response rather than a uniform effect across all outcomes. This pattern is compatible with previous evidence that nature exposure can support both physiological and psychological outcomes [47,48,49].

The physical outcomes showed a differentiated rather than uniform pattern. The clearest group × time effect was observed for TUG (η^2^p = 0.43), followed by grip strength and eyes-closed sway velocity (η^2^p = 0.23 for both). Post-intervention group differences were evident for TUG, pinch strength, and both eyes-closed balance measures, whereas grip strength and eyes-open sway area showed significant interactions without significant post-intervention differences, and eyes-open sway velocity showed no significant interaction. This pattern indicates that the largest observed physical effect was for functional mobility and that postural-control differences were more evident when visual input was unavailable. Notably, the SVEC interaction reflected both improvement in the CMTP group and deterioration in the control group, whereas the other physical outcomes were largely characterized by improvement in the CMTP group with relative stability in the control group. These findings are consistent with evidence that resistance- and strength-based exercise supports muscular performance, gait, and balance [50,51,52], and that core-stabilization exercise can enhance trunk control and postural stability [53,54,55,56,57]. The varied motor and loading demands of coastal walking, elastic-band resistance exercise, and core- and lower-limb activities offer one possible explanation for these changes [58,59]. Thus, the five-day CMTP was associated with selective functional changes, particularly in mobility and eyes-closed balance control.

The self-reported psychological and health-related quality-of-life outcomes showed a broader and more uniform pattern than the performance-based physical measures. All SF-36 summary and subscale scores, PSS-10, and BDI-II showed significant group × time interactions and post-intervention between-group differences. The largest observed group × time interaction effect size was for BDI-II (η^2^p = 0.50), followed by bodily pain (η^2^p = 0.45) and the mental component summary (η^2^p = 0.42); vitality and mental health also showed comparatively large interaction effects (η^2^p = 0.39 for both). These effect-size patterns suggest that the immediate response to the five-day program was most evident in perceived depressive symptoms, bodily pain, and mental health-related quality of life. Prior evidence links mindfulness-based practice to attentional and emotional regulation, body awareness, and relaxation [60,61,62,63,64,65]. Separate evidence associates nature exposure and physical activity with restorative experiences and favorable outcomes in stress, mood, and quality of life [66,67,68,69,70]. These factors provide a plausible context for the consistent short-term changes observed across the self-reported psychological and health-related quality-of-life outcomes.

Previous modality-specific studies provide a useful context for interpreting the present response profile. Studies of fall-prevention and core-stabilization programs have primarily reported changes in balance, gait, and quality of life [71,72], whereas studies of Nordic walking and Mat Pilates have demonstrated concurrent physical, psychological, or autonomic responses [73,74]. The present findings are broadly consistent with this literature in showing concurrent changes across functional and self-reported domains.

Variation across outcome domains may reflect both the constructs assessed by the outcome measures and the task specificity of physical performance. Randomized studies in menopausal and postmenopausal women have shown that mindfulness-based interventions can produce changes across several self-reported domains, including quality of life, stress, and depressive symptoms [19,75]. In contrast, evidence from peri- and early postmenopausal women indicates that exercise-related balance changes vary according to the exercise modality and the specific balance outcome assessed [18]. Balance-training research in healthy adults likewise suggests that improvement is often greatest in the trained task, with limited transfer to dissimilar balance tasks [76]. In the present study, this distinction was reflected in consistent changes across the SF-36, PSS-10, and BDI-II, whereas the clearest physical effects were concentrated in TUG and eyes-closed balance. Overall, the observed profile reflects broad changes across self-reported health outcomes accompanied by selective, task-dependent changes in physical performance.

This study has several limitations. Although the sample exceeded the a priori requirement, it remained relatively small and included relatively healthy, urban-dwelling women from one geographic region, which may limit generalizability. No follow-up assessment was conducted, so the persistence of the observed changes remains unknown. Because the CMTP combined physical activity, mindfulness meditation, and nature-based activities, the individual contributions of these components and the coastal environment could not be separated. The psychological outcomes were self-reported immediately after the program and therefore reflect immediate perceived responses. In addition, the usual-routine control condition did not match the intervention for attention, expectation, novelty, social interaction, instructor contact, or changes in daily routine, and allocation was known to participants and assessors. Bonferroni adjustment was applied within the simple-effect comparison families but not across outcome measures; therefore, findings for the secondary outcomes and individual SF-36 component and domain scores should be interpreted as exploratory. Future studies should use larger and more diverse samples, active comparators, component-specific or multi-arm designs, and follow-up assessments at 3 and 6 months.

## 5. Conclusions

Relative to the usual-routine control condition, the five-day supervised CMTP was associated with immediate changes in the prespecified primary outcome measures of functional mobility and multidimensional health-related quality of life. Exploratory analyses suggested selective changes in additional performance-based physical outcomes and a broader pattern across individual SF-36 domains and other self-reported outcomes. These findings support further evaluation of brief multicomponent coastal health programs for postmenopausal women. Future studies using active comparators, component-specific designs, and follow-up assessments are needed to determine the persistence of these changes and the relative contributions of the program components and coastal context.

## Figures and Tables

**Table 1:** Daily schedule of the five-day Combined Marine Treatment Program.

Time	Day 1	Day 2	Day 3	Day 4	Day 5
07:00–08:00		Breakfast and self-care	Breakfast and self-care	Breakfast and self-care	Breakfast and self-care
08:00–09:00		Coastal walking	Coastal walking	Coastal walking	Coastal walking
09:00–10:00	Orientation	Beach-based body recovery exercise	Beach-based body recovery exercise	Beach-based body recovery exercise	Beach-based body recovery exercise
10:00–11:00					
11:00–12:00					
12:00–13:00	Lunch and break	Lunch and break	Lunch and break	Lunch and break	Lunch and break
13:00–14:00					Program completion
14:00–17:00	Coastal sensory exploration and meditation	Visit to Chollipo Arboretum	Coastal sensory exploration and meditation	Visit to the Sinduri Coastal Sand Dune	
17:00–18:00					
18:00–19:00	Dinner	Dinner	Dinner	Dinner	
19:00–20:00	Mindfulness meditation	Mindfulness meditation	Mindfulness meditation	Mindfulness meditation	
20:00–21:00					
21:00–07:00	Rest and sleep	Rest and sleep	Rest and sleep	Rest and sleep	

**Table 2:** General characteristics of participants.

Variable	CMTP (*n* = 31)	CG (*n* = 31)	*p*-Value
Age (years)	58.13 ± 7.11	57.47 ± 6.86	0.710
40–49 (*n*)	3	4	
50–59 (*n*)	14	13	0.914
60–69 (*n*)	14	14	
Body height (cm)	154.97 ± 4.55	153.99 ± 4.29	0.389
Body weight (kg)	59.08 ± 6.51	58.69 ± 6.32	0.813
Body mass index (kg/m^2^)	24.62 ± 2.71	24.76 ± 2.77	0.842
Employment status			
Working (*n*)	20	21	0.788
Non-working (*n*)	11	10	
Education level			
High-school graduate or lower (*n*)	15	14	
College graduate (*n*)	11	12	0.962
Graduate student or higher (*n*)	5	5	

Values are presented as mean ± standard deviation or *n*. *p*-values were obtained using independent-sample *t*-tests for continuous variables and chi-square tests for categorical variables. CMTP, Combined Marine Treatment Program group; CG, control group.

**Table 3:** Changes in performance-based physical function outcomes after the program.

Variable	Group	Baseline	Post- Intervention	Within-Group *p*	Between-Group *p*	Group × Time *p*	η^2^p
TUG (s)	CMTP	7.97 ± 0.91	6.73 ± 0.85	<0.001	<0.001	<0.001	0.43
	CG	8.00 ± 0.96	7.95 ± 0.91	0.299			
Grip (kg)	CMTP	20.11 ± 5.03	22.05 ± 4.50	<0.001	0.115	<0.001	0.23
	CG	20.32 ± 4.73	20.13 ± 4.97	0.211			
Pinch (kg)	CMTP	5.41 ± 1.89	6.50 ± 0.83	<0.001	<0.001	<0.001	0.17
	CG	5.16 ± 1.48	5.28 ± 1.70	0.213			
Static Balance							
SAEO (cm^2^)	CMTP	1.24 ± 0.82	0.97 ± 0.45	0.011	0.070	0.003	0.14
	CG	1.13 ± 0.41	1.20 ± 0.50	0.113			
SVEO (cm/s)	CMTP	2.36 ± 0.29	2.21 ± 0.20	0.007	0.089	0.076	0.05
	CG	2.28 ± 0.52	2.33 ± 0.33	0.583			
SAEC (cm^2^)	CMTP	1.47 ± 0.84	1.24 ± 0.71	<0.001	0.019	0.002	0.14
	CG	1.66 ± 0.90	1.75 ± 0.93	0.266			
SVEC (cm/s)	CMTP	2.62 ± 0.43	2.25 ± 0.48	<0.001	0.002	<0.001	0.23
	CG	2.41 ± 0.51	2.70 ± 0.61	0.027			

Values are presented as mean ± standard deviation. Pairwise *p*-values are Bonferroni-adjusted; group × time *p*-values and η^2^p are from the mixed repeated-measures analysis of variance. CMTP, Combined Marine Treatment Program group; CG, control group; TUG, Timed Up and Go test; SA, sway area; SV, sway velocity; EO, eyes open; EC, eyes closed; η^2^p, partial eta squared.

**Table 4:** Changes in SF-36, PSS-10, and BDI-II scores after the program.

Variable	Group	Baseline	Post- Intervention	Within-Group *p*	Between-Group *p*	Group × Time *p*	η^2^p
**SF-36**							
**PCS**	CMTP	44.20 ± 20.74	67.43 ± 17.48	<0.001	<0.001	<0.001	0.38
	CG	43.49 ± 20.60	44.23 ± 20.58	0.057			
PF	CMTP	62.74 ± 23.11	77.81 ± 17.13	<0.001	0.005	<0.001	0.19
	CG	61.73 ± 22.49	62.82 ± 23.05	0.115			
RP	CMTP	21.27 ± 28.64	53.10 ± 32.15	<0.001	<0.001	<0.001	0.29
	CG	20.67 ± 27.24	21.25 ± 28.46	0.281			
BP	CMTP	48.56 ± 23.16	75.73 ± 17.20	<0.001	<0.001	<0.001	0.45
	CG	47.90 ± 22.83	48.62 ± 23.04	0.282			
GH	CMTP	44.22 ± 20.59	63.08 ± 17.76	<0.001	<0.001	<0.001	0.30
	CG	43.67 ± 21.91	44.23 ± 20.38	0.435			
**MCS**	CMTP	50.41 ± 18.42	74.98 ± 13.64	<0.001	<0.001	<0.001	0.42
	CG	49.90 ± 18.66	50.46 ± 18.34	0.194			
VT	CMTP	48.36 ± 16.47	75.60 ± 13.32	<0.001	<0.001	<0.001	0.39
	CG	47.64 ± 16.24	48.42 ± 16.44	0.272			
SF	CMTP	53.97 ± 21.70	72.83 ± 17.29	<0.001	<0.001	<0.001	0.20
	CG	53.42 ± 21.86	54.01 ± 21.58	0.410			
RE	CMTP	39.96 ± 37.55	71.13 ± 36.27	<0.001	0.002	<0.001	0.23
	CG	40.19 ± 37.15	39.98 ± 37.46	0.778			
MH	CMTP	59.34 ± 14.75	80.38 ± 13.91	<0.001	<0.001	<0.001	0.39
	CG	58.37 ± 14.98	59.41 ± 14.70	0.147			
**PSS-10**	CMTP	18.21 ± 4.62	11.52 ± 4.86	<0.001	<0.001	<0.001	0.36
	CG	17.77 ± 4.12	18.24 ± 4.63	0.105			
**BDI-II**	CMTP	17.11 ± 8.59	7.35 ± 5.05	<0.001	<0.001	<0.001	0.50
	CG	16.68 ± 8.33	17.15 ± 8.61	0.068			

Values are presented as mean ± standard deviation. Pairwise *p*-values are Bonferroni-adjusted; group × time *p*-values and η^2^p are from the mixed repeated-measures analysis of variance. CMTP, Combined Marine Treatment Program group; CG, control group; SF-36, Short Form-36 Korean version; PCS, Physical Component Summary; PF, Physical Functioning; RP, Role Functioning Physical; BP, Bodily Pain; GH, General Health; MCS, Mental Component Summary; VT, Vitality; SF, Social Functioning; RE, Role Functioning Emotional; MH, Mental Health; PSS-10, 10-item Perceived Stress Scale; BDI-II, Beck Depression Inventory-II; η^2^p, partial eta squared.

## Data Availability

The data presented in this study are available on request from the corresponding author due to privacy and ethical restrictions.

## Abbreviations

The following abbreviations are used in this manuscript:

APC	Article processing charge
BDI-II	Beck Depression Inventory-II
BP	Bodily Pain
CG	Control group
CMTP	Combined Marine Treatment Program
CONSORT	Consolidated Standards of Reporting Trials
CRIS	Clinical Research Information Service
EC	Eyes closed
EO	Eyes open
GH	General Health
ICC	Intraclass correlation coefficient
MCS	Mental Component Summary
MH	Mental Health
MSIT	Ministry of Science and ICT
NRF	National Research Foundation of Korea
PCS	Physical Component Summary
PF	Physical Functioning
PSS-10	10-item Perceived Stress Scale
RCT	Randomized controlled trial
RE	Role Functioning Emotional
RP	Role Functioning Physical
SA	Sway area
SAEC	Sway area under eyes-closed condition
SAEO	Sway area under eyes-open condition
SF	Social Functioning
SF-36	Short Form-36 Korean version
SPSS	Statistical Package for the Social Sciences
SV	Sway velocity
SVEC	Sway velocity under eyes-closed condition
SVEO	Sway velocity under eyes-open condition
TUG	Timed Up and Go test
VT	Vitality
